# Factors Associated with Uptake of Visual Inspection with Acetic Acid (VIA) for Cervical Cancer Screening in Western Kenya

**DOI:** 10.1371/journal.pone.0157217

**Published:** 2016-06-16

**Authors:** Elkanah Omenge Orang’o, Juddy Wachira, Fredrick Chite Asirwa, Naftali Busakhala, Violet Naanyu, Job Kisuya, Grieven Otieno, Alfred Keter, Ann Mwangi, Thomas Inui

**Affiliations:** 1 Academic Model Providing Access to Healthcare (AMPATH), Eldoret, Kenya; 2 Department of Reproductive Health, School of Medicine, College of Health Sciences, Moi University, Eldoret, Kenya; 3 Department of Pharmacology/Oncology, School of Medicine, College of Health Sciences, Moi University, Eldoret, Kenya; 4 Department of Behavioral Sciences, School of Medicine, College of Health Sciences, Moi University, Eldoret, Kenya; 5 Department of Medicine, School of Medicine, Indiana University, Indianapolis, United States of America; 6 Regenstrief Institute, Inc. Indianapolis, United States of America; Bharathidasan University, INDIA

## Abstract

**Purpose:**

Cervical cancer screening has been successful in reducing the rates of cervical cancer in developed countries, but this disease remains the leading cause of cancer deaths among women in sub-Saharan Africa. We sought to understand factors associated with limited uptake of screening services in our cervical cancer-screening program in Western Kenya.

**Participants and Methods:**

Using items from a previously validated cancer awareness questionnaire repurposed for use in cervical cancer and culturally adapted for use in Kenya, we interviewed 2,505 women aged 18–55 years receiving care in gynecology clinics or seeking other services in 4 health facilities in Western Kenya between April 2014 and September 2014. We used logistic regression modeling to assess factors associated with uptake (or non-uptake), associated odds ratios (ORs) and the 95% confidence intervals (95% CI).

**Results:**

Only two hundred and seventy-three women out of 2505 (11%) accepted VIA cervical cancer screening. Knowledge of just how women are screened for cervical cancer was significantly associated with reduced uptake of cervical cancer screening (OR: 0.53; CI 0.38–0.73) as was fear that screening would reveal a cancer (OR 0.70; CI 0.63–0.77), and reliance on prayer with the onset of illness (OR 0.43; CI 0.26–0.71). Participants who thought that one should get cervical cancer screening even if there were no symptoms were more than twice as likely to accept cervical cancer screening (OR 2.21; 95% CI 1.24–3.93). Older patients, patients living with HIV and women who do not know if bleeding immediately after sex might be a sign of cervical cancer were also more likely to accept screening (OR 1.03, CI 1.02–1.04; OR 1.78, CI 1.01–3.14; OR 2.39, CI 1.31–4.39, respectively).

**Conclusions:**

In our population, a high percent of women knew that it is appropriate for all women to get cervical cancer screening, but only a small proportion of women actually got screening. There may be an opportunity to design educational materials for this population that will not only encourage participation in cervical cancer screening but also remediate misconceptions. The discussion illustrates how our findings could be used in such an effort.

## Introduction

Cervical cancer is the leading cause of cancer deaths among women in Africa [1]. Over 80% of worldwide invasive cervical cancers occur in developing world populations, largely as a result of the challenges in establishing effective screening programs [2]. The World Health Organization estimates that only about 5 percent of women have been screened for cervical cancer in resource-poor countries, compared to 40–50 percent in the developed world [3]. Kenya currently has very limited cervical cancer screening [1, 4].

Cervical cancer has the highest incidence of any malignancy among women in Kenya and is the second most common cause of death [1]. A large portion of this disease burden could be reduced by the administration of effective screening programs [5].

Like Cervical Intraepithelial Neoplasia (CIN), invasive cervical cancer is also more common in human immunodeficiency virus (HIV)-infected women and has been considered an AIDS-defining illness since 1993[4,5]. Published data indicate that HIV-infected women present with invasive cervical cancer 10 to 15 years earlier than HIV-negative women, manifest with more advanced disease and have a poorer prognosis compared with HIV-negative counterparts.

Progression of cervical cancer in these patients is also more rapid and unfortunately often refractory to therapy, with high recurrence rates [4,5,6].

Pap test screening has been successful in reducing the rates of cervical cancer in developed countries. [7] However, the many logistical prerequisites for a good Pap smear-based program have been difficult to implement in developing countries. The Pap test requires preparation of high-quality smears, well-trained experienced personnel, and internal and external control mechanisms to reach a high percentage of the population. [8,9]

Given these Pap test constraints, alternative methods such as Visual inspection with acetic Acid (VIA) of cervical screening have been tested in resource-limited countries [8–12]. VIA is a visualization of the cervix after application of acetic-acid to identify abnormal areas. Single-visit programs of VIA and immediate treatment (cryotherapy) have been shown to decrease the prevalence of high-grade cervical cancer precursor lesions [13–15].

In our previous work, we assessed the accuracy of visual inspection with acetic acid (VIA) versus conventional Pap smear as a screening tool for cervical intraepithelial neoplasia/cancer among HIV infected women in our service area population. In that study, sensitivity, specificity, PPV, and NPV of VIA was 69.6%, 51.0%, 38.6%, and 79.1%, respectively, for CIN 2 or worse lesions. While these performance characteristics are clearly not ideal, in our facilities, Pap smear cytology was not clearly superior [12]. As a consequence, we have adopted VIA as our standard for screening. A positive is followed by cervical cryotherapy and follow-up colposcopy. False positives do not recur as VIA lesions.

### Knowledge and Attitudes toward cervical cancer screening

Understanding what women know and think about testing is of particular importance in cervical cancer, which is an entirely preventable disease, one that is amenable to both primary and secondary prevention strategies. Previous studies across the world have demonstrated that knowledge of HPV, HPV vaccination, cervical screening, and cervical cancer risk factors is extremely poor [16–20]. Intention to receive the HPV vaccine has also been significantly associated with knowledge of cervical screening and cervical cancer risk factors [21–23]. Health promotion efforts need to focus on understanding women's knowledge of risk factors and enhancing their perceived health control by providing more information on the link between screening and early detection with lower incidence rates and mortality from cervical cancer.

### AMPATH Cervical Cancer Screening Program (ACCSP)

The Academic Model Providing Access to Healthcare (AMPATH) is a consortium of universities in North America that partner with Moi University, Moi Teaching and Referral Hospital in Eldoret, Kenya, and the Ministry of Health of Kenya to deliver health care, education/training, and research. Services provided by AMPATH involve urban and rural medical care for HIV and chronic non- communicable diseases, including cervical cancer. The AMPATH cervical cancer screening and prevention program (ACCSP) began in 2008 with research funding from the Fogarty International Center.

In 2011, 6427 women were screened for cervical cancer with VIA at four AMPATH sites: Eldoret, Turbo, Mosoriot, and Webuye. We do not know how many women were offered but declined to be screened, but 17% of those screened were found to be VIA positive and referred for follow-up management.;30% of the clients were referred for other management (colposcopy or biopsy-confirmed diagnosis thus requiring a surgical procedure (LEEP or hysterectomy), and 22% were lost to follow-up. By the end of February 2015, a total of 32,585 women had been screened for cervical cancer in 9 sites in Western Kenya.

We do not understand why many women who regularly come for their HIV care appointment do, or do not, utilize cervical cancer screening services. We, therefore, conducted a study at four cervical cancer-screening sites to help us understand factors that may be associated with uptake of screening services in our program. The sites included two gynecology clinics based at Moi Teaching and Referral Hospital in Eldoret and another two in rural health facilities (Turbo Health Centre and Webuye County Hospital).

The aim of this study was to identify factors associated with uptake of cervical cancer screening among women seeking care at gynecology clinics in western Kenya.

## Materials and Methods

### Study design

This was a prospective study conducted in the above-cited four clinics in the AMPATH catchment area. Convenience sampling method was used to recruit and consent 2,505 women aged 18–55 years in patients receiving care in gynecology clinics. We used questionnaires and cancer screening records for data sources.

Moi Teaching and Referral Hospital Institutional Research and Ethics Committee (IREC) as well as the Indiana University Institutional Research Board (IRB) committee approved the study.

### Study procedures

Research assistants with at least a secondary level of education were recruited and trained to facilitate data collection. These research assistants approached all women in the waiting bay as they waited to be attended to by clinicians. Patients were informed about the study and invited to participate. A research assistant directed those women who agreed to participate in the study to a private room. A recruitment instrument [S1 Appendix] was administered to determine eligibility. Those who met the eligibility criteria were requested to provide a written consent for their participation in the study. An interviewer-administered questionnaire with both structured and open-ended questions [S2 Appendix] was administered to participants in either English or Swahili. At the end of the survey, the participants were invited to undergo VIA screening. Those who agreed to the screen were directed to the cervical cancer screening room. A unique identifier was given to participants and at the end of each day the research assistant checked the attendance list to determine whether actual cervical cancer screening occurred. The list of participants only had unique identifiers and not participants' names.

### Data analysis

Data analysis was done using STATA version 13 SE, College Station, Texas 77845 USA. Categorical variables were summarized as frequencies and the corresponding percentages. Gaussian assumptions for the continuous variables were assessed empirically using Shapiro-Wilk test. Since the variables violated the Gaussian assumptions they were summarized as median and the corresponding interquartile range. We assessed association between categorical variables using Person's Chi Square test. The association between continuous variables and categorical (binary) variables was assessed using two-sample Wilcoxon rank-sum test.

## Results

### Demographics of study participants

A total of 2505 women were recruited to the study. Two hundred and seventy- three women (11%) accepted VIA screening. Basic demographic characteristics of the enrolled sample are displayed in Table 1.

**Table 1:** Demographic and socio-economic characteristics.

				Uptake of screening	
Variable	Sample Size	Levels	Total (2505,100%)	^†^No (2232,89%)	^†^Yes (273,11%)
^w^Age per unit increase (years)	2502		38 (31–45)	38(31–45)	39(31–46)
^w^Household members	2501		4(3–6)	4(3–6)	4(3–5)
^c^Marital status	2504	Married	1080(43%)	965(43%)	115(42%)
		Other	1424(57%)	1266(57%)	158(58%)
^w^Parity	2414		3(2–5)	3(2–5)	3(2–5)
^c^Site	2505	AMPATH	1141(46%)	1017(46%)	124(45%)
		MTRH	231(9%)	200(9%)	31(11%)
		Turbo	519(21%)	470(21%)	49(18%)
		Webuye	614(25%)	545(24%)	69(25%)
^w^Travel time to the facility (Minutes)	2503		45(30–80)	45(30–80)	45(30–60)
^c^Means of transport to the facility	2503	Walking	277 (11%)	251 (11%)	26 (10%)
		Public (Boda boda, Matatu, Bus)	2195 (88%)	1949 (87%)	246 (90%)
		Private	31 (1.2%)	30 (1.4%)	1 (0.4%)
^c^Education level	2504	None	131(5%)	118(5%)	13(5%)
		Primary	1004(40%)	892(40%)	112(41%)
		Secondary	988(39%)	879(39%)	109(40%)
		College/University	381(15%)	342(15%)	39(14%)
^c^Work type	2503	Unemployed	754(30%)	671(30%)	83(30%)
		Short term/casual	465(19%)	424(19%)	41(15%)
		Employed	414(17%)	366(16%)	48(18%)
		Self-employed	870(35%)	769(34%)	101(37%)
^c^Income (Kshs. per month per household)	1652	<4000	511(31%)	440(30%)	71(38%)
		4001–10999	526(32%)	473(32%)	53(29%)
		11000–20999	403(24%)	367(25%)	36(19%)
		≥21000	212(13%)	187(13%)	25(14%)

^c^ Pearson’s Chi Square test;

^w^ two-sample Wilcoxon rank-sum test

^†^Between groups comparisons were not statistically significant; all p’s > .05

Comparing women who accepted VIA screening with those who did not, there were no statistical differences in the patients' demographic characteristics (e.g. age, education level, marital status, household size, work type, household income, and usual means of transportation (see Table 1 footnotes for tests and significance statement). Clinical and care-seeking characteristics including the number of sex partners, parity, cigarette smoking, use of herbalists, use of private versus government clinics, clinic site, travel time to clinic and use of clinic for regular health checkups were also not different among VIA acceptors versus non-acceptors. Finally, items dealing with various barriers to accessing screening, including expense, fear, embarrassment, and the disapproval of others showed no statistically significant difference between acceptors and non-acceptors.

The comparison of time required for use of the different means of transport to clinic revealed that the participants who used public means of transport spent a significantly longer time, median (IQR): 45.0 (30.0, 80.0) minutes compared to those who walked, 30.0 (IQR: 20.0, 60.0) minutes, and those who used private vehicles, 30.0 (IQR: 20.0, 30.0) minutes, p = 0.0001. Transport differences, however, did not distinguish between those who accepted and those who declined VIA cervical screening.

### Common knowledge about cervical cancer

In additional bivariate analyses of knowledge differences between acceptors versus non-acceptors (see Table 2). Knowledge of symptoms of cervical cancer showed that a significantly higher proportion not knowing whether bleeding immediately after sex was a sign of cervical cancer increased acceptance of cervical cancer screening, 86(32%) vs. 551(25%). Those who thought that bleeding immediately after sex was not a sign of cervical cancer also had higher chances of accepting cervical cancer screening, 271(12%) vs. 21(8%) vs. p = 0.029. There was a significantly higher proportion among VIA acceptors among those who thought that one would need to go for cervical cancer screening even if there were no symptoms, 223(82%) vs. 1695(76%), p = 0.049.

**Table 2:** Symptoms of cervical cancer.

				Uptake of screening		
Variable	Sample size	Levels	Total (2505,100%)	No (2232,89%)	Yes (273,11%)	P
Bleeding immediately after sex is a sign of cervical cancer	2498	No	292(12%)	271(12%)	21(8%)	0.029
		Yes	1229(49%)	1096(49%)	133(49%)	
		Maybe	340(14%)	307(14%)	33(12%)	
		Don’t know	637(26%)	551(25%)	86(32%)	
Think one would need to go for screening even if there were no symptoms	2496	No	231(9%)	218(10%)	13(5%)	0.049
		Yes	1918(77%)	1695(76%)	223(82%)	
		Maybe	144(6%)	130(6%)	14(5%)	
		Don’t know	203(8%)	180(8%)	23(8%)	

Analyses assessing perceived individual susceptibility to, and perceived severity of, cervical cancer, revealed no significant differences between acceptors and non-acceptors of screening. This included responses to questions about the likelihood of getting cervical cancer in one's lifetime, family history, and the impact of cervical cancer on sexual relationships, fertility, and longevity.

### Knowledge about Cervical cancer screening

Finally, some significant differences between acceptors and non-acceptors emerged in bivariate analyses of items assessing knowledge about the process of cervical cancer screening itself (Table 3). While acceptors and non-acceptors did not differ in views of the risk of the screening procedure for their sexual health or how much time the procedure takes, there were several other differentiating points of knowledge, including knowing how women are screened and recommendations to members of patients' families. Nearly significant were differences in knowledge of frequency of screening and not knowing if screening might be painful.

**Table 3:** Knowledge about cervical cancer screening.

				Uptake of screening		
Variable	Sample size	Levels	Total (2505,100%)	No (2232,89%)	Yes (273,11%)	P
Know how women are screened?	2493	No	1254(50%)	1087(49%)	167(62%)	<0.0001
		Yes	997(40%)	928(42%)	69(25%)	
		Maybe	11(0.4%)	10(0.5%)	1(0.4%)	
		Don’t know	231(9%)	197(9%)	34(13%)	
Frequency of cervical cancer screening	2496	Any time	187(7%)	165(7%)	22(8%)	0.06
		Monthly	149(6%)	131(6%)	18(7%)	
		Every 6 months	377(15%)	336(15%)	41(15%)	
		Every year	653(26%)	569(26%)	84(31%)	
		Every 3 years	686(27%)	633(28%)	53(20%)	
		Don’t know	444(18%)	391(18%)	53(20%)	
Think that cervical cancer screening is painful	2495	No	1324(53%)	1199(54%)	125(46%)	0.05
		Yes	453(18%)	401(18%)	52(19%)	
		Maybe	148(6%)	126(6%)	22(8%)	
		Don’t know	570(23%)	497(22%)	73(27%)	
Would tell your family member to be screened?	2494	No	101(4%)	96(4%)	5(2%)	0.002
		Yes	2144(86%)	1912(86%)	232(85%)	
		Maybe	125(5%)	100(5%)	25(9%)	
		Don’t know	124(55)	114(5%)	10(4%)	

Knowledge of how women are screened for cervical cancer was significantly associated with reduced rate of uptake for cervical cancer screening, 69(25%) vs. 928(42%), p<0.0001. A significantly lower proportion of those who would not tell a family member to go for cervical cancer screening was seen among those who were screened for cervical cancer compared to those who were not screened, 5(2%) vs. 96(4%), p = 0.002.

### Determinants of uptake of cervical cancer screening

Given the several differences between acceptors and non-acceptors of VIA screening documented by our bivariate analyses, the definitive analysis of predictors of VIA uptake must be a multivariable analysis that adjusts for confounding among independent variables. The results of the logistic regression predicting VIA acceptance (dependent variable) are displayed in Table 4.

**Table 4:** Factors associated with uptake of cervical cancer screening in multivariate model.

	Uptake of screening	
		Odds Ratio (95% CL)
Age per unit increase (years)		**1.03 (1.02, 1.04)**
Currently living with HIV	Yes vs. †No	**1.78 (1.01, 3.14)**
Bleeding immediately after sex is a sign of cervical cancer	Yes or Maybe vs. †No	1.57 (0.98, 2.54)
	Don’t Know vs. †No	**2.39 (1.31, 4.39)**
Know how women are screened for cervical cancer	Yes or Maybe vs. †No	**0.53 (0.38, 0.73)**
	Don’t Know vs. †No	0.84 (0.68, 1.04)
Know where to go for cervical cancer screening	Yes vs. †No	**0.51 (0.32, 0.80)**
The first thing that one does when they get sick	Go for Prayers (Yes vs. †No)	**0.43 (0.26, 0.71)**
Likely to get cancer sometimes in lifetime	Yes or Maybe vs. †No	1.20 (0.69, 2.07)
	Don’t Know vs. †No	**1.90 (1.36, 2.64)**
Frequency of screening for cervical cancer	Monthly vs. †Anytime	1.62 (0.94, 2.79)
	Every 6 months vs. †Anytime	**0.47 (0.26, 0.84)**
	Every year vs. †Anytime	0.84 (0.42, 1.66)
	Every 3 years vs. †Anytime	**0.51 (0.31, 0.86)**
	Don’t Know vs. †Anytime	**0.49 (0.30, 0.82)**
Afraid to have cervical cancer screening for fear of having one	Yes or Maybe vs. †No	**0.70 (0.63, 0.77)**
Would be screened for cervical cancer even if one had to pay	Yes or Maybe vs. †No	**1.47 (1.11, 1.94)**
	Don’t Know vs. †No	**0.28 (0.10, 0.73)**
	**Sample size: 850**	

^†^ Reference group

This analysis reveals several significant associations with acceptance/non-acceptance of screening. Patients more likely to accept screening included: older patients, patients living with HIV and women who do not know if bleeding immediately after sex might be a sign of cervical cancer. Those who said that they would go for cervical screening even if they had to pay, and those who do not know if they are likely to get this cancer in their lifetime were likely to accept screening. Patients less likely to take screening included women who knew how cervical cancer screening was done, those who knew where to go for cervical cancer screening, those who reported prayer as the first health-seeking option whenever they fall sick, women who thought that screening is recommended only every three years and women who reported that they were afraid to be screened because it might reveal that they had the cancer. Household income, the number of household members, parity, and a number of lifetime sexual partners were not associated with uptake of cervical cancer screening but were controlled for in this analysis.

## Discussion

In this study, the only one of its kind in sub-Saharan Africa, we use a survey of cervical cancer awareness to explore a paradox—reported favorable inclinations to obtaining cervical cancer screening with very low rates of actual screening. We hope that the approach we have taken to using output from our survey, exploring these data for associations with obtaining screening, taking special note of item responses that could be integrated into educational programming, and attending to cultural sensitivity issues will be contemplated by others as a path to improving cervical cancer screening in their own populations.

From our study findings, a vast majority of gynecology clinic patients (81%) in our setting believe that all women need cervical cancer screening. Only 11% of the women participating in the study, however, actually elected to get VIA examination done at their study visits. Even after our survey and analyses, a complete understanding of this disinclination to get screened remains elusive. Since these women were approached to participate in the study while they had come to seek other health care services, it is possible that the one-day screening event did not provide adequate time for them to seek the services, among other potential barriers. There may also be a need for education and ‘marketing' VIA screening at the date of clinic registration to emphasize the need for cervical cancer screening for all women. Many studies have revealed that the commonest barrier to uptake of cervical cancer screening is a feeling of anxiety concerning the possibility of receiving positive screening results. Yao Jia et al. in their study on knowledge to cervical cancer and barriers to screening, for example, reported no symptoms/no discomfort, lack of awareness of benefits of cervical cancer screening, fear of screening procedure and possibility of diagnosing incurable cervical cancer as among barriers to uptake of screening services by Chinese women [24]. Closer to our service area, in sub-Saharan Africa, confusion about whether the screening test has a diagnostic purpose only or whether it can be linked to early treatment and cure, as well as ignorance about the causes of cervical cancer, cultural constructs/beliefs about the illness, economic factors, domestic gender power relations (e.g. husband unwillingness to fund screening), alternative traditional sources of reproductive health information (family members or traditional healers), and unfriendly health care service providers can be barriers to accepting screening [25,26]. Other impediments to accepting screening have included culture-based reluctance to ‘show my private parts,’ ‘opening my legs’ for an exam and in some situations gender preferences for examination by practitioners of the same or different gender [25,27].

We have uncovered some information in our survey that might be used to persuade women to accept screening. Our findings indicate women who report that they are HIV-positive in this clinic attendee population are more likely to be screened for cervical cancer than their HIV-negative counterparts. This is good news since studies have shown, like Cervical Intraepithelial Neoplasia (CIN), invasive cervical cancer is also more common in HIV-infected women, and has been considered an AIDS-defining illness. While self-reported HIV was associated with accepting screening, not all those who acknowledge living with HIV got screened. Education might put useful emphasis on the need for universal screening in this higher-risk group. In HIV-negative women, however, since cervical cancer is caused by a sexually transmitted virus and is associated with HIV positivity, HIV stigma may be one of the potential barriers to the utilization of cervical cancer screening programs. A study by Joelle et al. found that cervical cancer stigma was highly correlated with HIV stigma (correlation coefficient 0.72) but was significantly lower in HIV-positive women (p < 0.001). Education might emphasize privacy, confidentiality, and that HIV screening need not be an intrinsic part of cervical cancer screening [25].

Our results also suggest that women who know how cervical cancer screening is done and where to go for screening are less likely to get screened in our setting. A possible explanation for this includes the fact that cervical cancer screening involves a pelvic examination, a procedure that may be perceived as invasive, possibly painful and culturally insensitive [25–28]. Some experience with pelvic exams and screening tests may be reassuring to women, since when women have accepted repeated pelvic exams, this concern diminishes [28]. Cultural acceptability concerns, however, while diminished are still present and highly variable. We also noted that whether a woman accepts screening herself is not associated with what recommendations she would make for her family members. This was a bit surprising and disappointing since we expect screened women to share their experiences with their relatives and, therefore, act as ambassadors for getting the service. It seems possible that general social norms may militate against discussion of vaginal/pelvic exams. Having education delivered by clinicians who are members of a woman's ethnic community when possible may be helpful in providing reassurance. Continuous community campaigns are needed to create more awareness about the importance and the procedure of cervical cancer screening. Several other beliefs may affect uptake of cervical screening services in this population that could be discussed with patients. Women whose answer was ‘no' to the question if bleeding immediately after sex is a sign of cervical cancer, for example, were less likely to get screened than those who report they don’t know. This response could be influenced by their knowledge or perhaps perceptions about how cervical cancer can present. Likewise, women who think screening frequency is anything except once a year are less likely to be screened. Women who believe it is needed only every three years are least likely to be tested and might have chosen not to seek the service on the day of our survey. Experts may actually differ on the recommended frequency of screening globally. While some health care systems recommend annual screening, it is actually acceptable to get screening less often if one is an average-risk woman. The American Cancer Society (ACS), American Society of Colposcopy and Cervical Pathology (ASCCP) and American Society of Clinical Pathology (ASCP) issued joint guidelines for cervical cancer screening in May 2012[29]. These recommendations indicate that screening should start at age 21 regardless of sexual history. Between age 21 and 29, screening is recommended every 3 years. Between age 30–65, HPV and cytology co-testing every 5 years is preferred, while cytology alone every 3 years is acceptable. Screening can be discontinued after age 65 after either three consecutive negative cytology tests within 10 years. HIV-Positive women represent an exception to the recommendation against starting screening before age 21. HIV-positive younger than 30 years are initially screened annually, but can undergo cytology testing every 3 years instead of annually if they have had three consecutive normal annual cytology tests [30]. In summary, community campaigns could be used to reassure these women that getting cervical cancer screening every three years is fine, but getting cervical cancer screening annually is also acceptable.

To promote participation, we note that women who are afraid of learning that they have cervical cancer are less likely to be screened. They may need to know that the most probable outcome of screening is a validation of good health and advice on how to avoid getting the condition in the first place (primary prevention). They also need to know that even if signs of early cancer are found, this condition can be quickly cured without risk to their sexual or reproductive well-being. Health promotion efforts need to focus on understanding women's knowledge of risk factors and enhancing their perceived health control by providing more information on the link between screening and early detection with lower incidence rates and mortality from cervical cancer. Finally, noting that uncertainty among our respondents about whether they would get screening if they had to pay for this service mediated strongly against accepting VIA, we would suggest disseminating more information about the actual charges for the service, especially if the charges are waived (e.g. among HIV+ populations in our setting).

### Limitations

This was a cross-sectional study using an interviewer-administered questionnaire. We, therefore, relied on study participants recalling certain past events. There may have been some recall or other respondent bias that affected the results of this study. This was a hospital/health facility-based survey, so our findings cannot be assumed to apply to community populations. The study, however, also has some strength. The sample size was large and to the best of our knowledge this is the first such study to be carried out among women in western Kenya. Findings from this study will, therefore, serve as a basis for future studies and may also be used to develop educational programming in our setting.

## Conclusion

This study has highlighted several important findings about the knowledge of women in western Kenya of cervical cancer and cervical cancer screening. It is encouraging to note that a high percentage of women know that it is necessary for all women to get cervical cancer screening. The small number of women who got cervical cancer screening is, however, worrisome. There could be many reasons for this discrepancy, including fear of the screening procedure, fear of a diagnosis of cervical cancer and its consequences [26–28]. There is an opportunity for designing appropriate educational materials for this population that will not only encourage their participation in cervical cancer screening activities but will also correct any misconceptions that may exist.

## Supporting Information

S1 Appendix Consent form. (DOC)

S2 Appendix Swahili consent form. (DOC)

S3 Appendix Questionnaire. (DOC)
