# Supplementary material for: Factors Associated with Uptake of Visual Inspection with Acetic Acid (VIA) for Cervical Cancer Screening in Western Kenya
Source: PLoS One. 2016 Jun 16;11(6):e0157217. doi: 10.1371/journal.pone.0157217 (PMC4911084; doi:10.1371/journal.pone.0157217)
Supplement: S1 Appendix — (DOC) [file pone.0157217.s001.doc]

**MOI TEACHING AND REFERRAL HOSPITAL/MOI UNIVERSITY INSTITUTIONAL**

**RESEARCH AND ETHICS COMMITTEE**

**INFORMED CONSENT STATEMENT FOR: Factors Associated with Uptake of Cervical Cancer Screening and Treatment: Application of the Health Belief Model**

**Introduction**

You are being invited to take part in a research study. Before you decide whether or not to take part, it is important for you to understand why the research is being done and what it will involve. Please take time to read the following information carefully.

**What is the purpose of the study?**

This is a survey to assess awareness of cancer of the cervix and factors that motivate women to come for screening and treatment. The study is being conducted by Dr Omenge Orango of Moi Teaching /Referral Hospital Oncology clinic and Professor Tom Inui of Indiana University-USA and the Walther team. The study is funded by the Walther grant.

The burden of cervical cancer is increasing in the developing world, which includes Kenya. After completing this survey you will be requested to go for cervical cancer screening that is offered at no extra cost to you.

The results of this survey will be used to develop better and more effective hospital communications and services to help increase the early diagnosis of cancer.

**Why have I been invited to take part?**

You have been randomly selected to participate in this study because you meet the inclusion criteria which have been explained to you.

**Do I have to take part?**

It is up to you to decide whether or not to take part. Taking part is voluntary. If you do decide to take part you will be asked to sign a consent form. Even after recruitment, you are still free to withdraw at any time and without giving a reason. You can decide to take part in the screening exercise but decline to take part in the survey.

**What would I have to do?**

If you agree to participate in this study, you will be asked some questions. You do not have to answer questions that you feel uncomfortable about. At the end of the survey, you will be invited to undergo screening. If you agree to screening you will be directed to the cervical cancer screening room.

If you decide to take part in the survey, answering the questions will take approximately 30 minutes to complete.

**WHAT ARE THE RISKS OF THE STUDY?**

**Loss of confidentiality but we will minimize this by ensuring that only members of the study team have access to your information and coding during data analysis.**

**WHAT ARE THE BENEFITS OF THE STUDY?**

There are no direct benefits to you for participating in this study, however information that you give may help the screening program develop better communication to other women and therefore detect cervical problems earlier.

WHAT ARE THE COSTS?

There will not be any additional cost to you for participating in this study

**WHAT ABOUT CONFIDENTIALITY?**

Efforts will be made to keep your personal information confidential. All details that can identify you will be removed before storing the data. We cannot guarantee absolute confidentiality. Your personal information may be disclosed if required by law.

Organizations that may inspect and/or copy your research records for quality assurance and data analysis include groups such as trained staff at Moi Teaching and Referral Hospital and trained staff from Indiana University

.

WHOM DO I CALL IF I HAVE QUESTIONS?

If you have any questions about the research study, you may contact Dr. Omenge Orango on 0722609132.

**Name of interviewer------------------------------------------Signature--------------Date------------**

**Name of Participant------------------------------------------Signature---------------Date------------**
