# Supplementary material for: Factors Associated with Uptake of Visual Inspection with Acetic Acid (VIA) for Cervical Cancer Screening in Western Kenya
Source: PLoS One. 2016 Jun 16;11(6):e0157217. doi: 10.1371/journal.pone.0157217 (PMC4911084; doi:10.1371/journal.pone.0157217)
Supplement: S2 Appendix — (DOC) [file pone.0157217.s002.doc]

**Fomu ya Kutoa Idhini**

**Utangulizi**

Unaalikwa kushiriki kwenye utafiti. Kabla ya kuamua kushiriki au kutoshiriki, ni vema kuelewa ni kwa nini utafiti huu unafanywa na itahusisha nini. Tafadhali chukua muda kusoma maelezo yafuatayo kwa uangalifu.

**Sababu ya utafiti ni nini?**

Huu ni ukaguzi unaotaka kujua ufahamivu wa hadhari ya saratani ya matiti, ishara na dalili, na pia kuchekecha saratani ya matiti. Utafiti huu unaongozwa na Daktari Omenge Orango wa Hospitali kuu ya rufaa na Mafunzo ya cliniki ya Oncologia na Profesa Tom Inui wa chuo kikuu cha Indiana, Marekani. Utafiti huu unagharamiwa na hiba la Walther.

Shida la saraatani ya mlango wa mfuko was uzazi inazidi kuongezeka kwenye nchi zinazoendelea ambazo inajumulisha Kenya. Huu utafiti unakuchekecha kwa saratani ya kizazi kwa sababu ugonjwa huu ukijulikana mapema, ni rahisi kutibu.

Majibu yatatumika kukuza mawasiliano na huduma iliyo bora na ya kufaa zaidi na pia kusaidia kuongezeka kwa kutambua saratani ikiwa bado ni mapema.

**Mbona nimealikwa kushiriki?**

Umechaguliwa bila utaratibu maalum kushiriki kwenye utafiti huu kwa sababu maelezo sawa na yale ya hali ya kushirikishwa ambayo ulikuwa umeelezwa.

**Lazima nishiriki?**

Ni jukumu lako mwenyewe kuamua kushiriki au kutoshiriki katika utafiti huu. Kushiriki ni kwa hiari. Ukiamua kushiriki kwenye utafiti huu, utaulizwa kuweka sahihi kwenye fomu ya kutoa idhini. Hata baada ya kujiandikisha, bado una huru wa kujiondoa wakati wowote na bila kupeana sababu. Unaweza kuamua kushiriki kwenye mazoezi ya kuchekecha lakini waweza kukataa kushiriki kwenye upelelezi.

**Ni nini nitakalofanya?**

Ukikubali kushiriki kwenye utafiti huu, utauliswa maswali kadhaa. Ukiamua kushiriki kwenye utafiti huu, kujibu maswali utachukua muda kama dakika 30 kumaliza.

**Hadhari zinazoambatana na kushiriki kwenye utafiti?**

**Kupotea kwa usiri lakini tutapunguza kabisa kwa kuhakikisha kuwa ni wafanyikazi wa uchunguzi huu peke yao wataweza kupata kuona recodi zako na tutaweka tarakimu maalum**

**Faida ya Utafiti huu?**

Hakuna faida ambayo utapata mwenyewe kwa kushiriki kwa utafiti huu. Hata hivyo kuna faida nyingi kwa habari utakayoitoa ambayo itasaidia kukuza mawasiliano na huduma iliyo bora na ya kufaa zaidi na pia kusaidia kuongezeka kwa kutambua saratani ikiwa bado ni mapema.

Gharama?

Hakutakuwa na gharama yoyote iliyo juu utakayopata unaposhiriki kwa utafiti huu.

**Na Usiri?**

Juhudi zozote zitawekwa ili maelezo yako binafsi yawe kwa usiri. Maelezo yoyote yanayokutambua yatatolewa kabla ya kuweka deta. Hatuwezi kuhakikisha usiri kikamilifu. Habari zako za kibinafsi zaweza kugunduliwa ikiwa inahitajika kisheria.

Miungano ambayo yanaweza kukagua na/au kunakili kumbukumbu zako za utafiti kwa nia ya kudhibiti sifa na imani na kuchanganua deta ni pamoja na makundi kama wafanyikazi waliosajiliwa wa Hospitali kuu ya rufaa na Mafunzo ya Moi na wafanyikazi waliosajiliwa wa Chuo Kikuu cha Indiana.

.

Nitawasiliana na nani nikiwa na swali?

Ukiwa na maswali yoyote kuhusu utafiti huu, unaweza kuwasiliana na Daktari Omenge Orango kwenya nambari ya simu 0722609132.

**Jina la Mhojaji------------------------------------------Sahihi--------------Tarehe------------**

**Jina la Mshiriki------------------------------------------Sahihi---------------Tarehe------------**
