# Supplementary material for: Factors Associated with Uptake of Visual Inspection with Acetic Acid (VIA) for Cervical Cancer Screening in Western Kenya
Source: PLoS One. 2016 Jun 16;11(6):e0157217. doi: 10.1371/journal.pone.0157217 (PMC4911084; doi:10.1371/journal.pone.0157217)
Supplement: S3 Appendix — (DOC) [file pone.0157217.s003.doc]

**ANNEX A.**

# Have you been screened for cervical cancer?

0 No

1 Yes

99 Not sure

# Do you have HIV?

0 No

1 Yes

99 Not sure

# Do you use tobacco?

# 0 No

#

# 1 Yes

#

# 99 Not sure

# Do you drink alcohol?

# 0 No

#

# 1 Yes

# 99 Not sure

# Do you have a history of intimate partner violence?

# 0 No

# 1 Yes

# 99 Not sure

# Have you been pregnant before? IF the answer is No or not sure skip to question 8.

# 0 No

# 1 Yes

# 99 Not sure

# How many previous pregnancies have you had?

# 0 none

# 2 1-5

# 3 more than 5

# 99 Unknown

# Have you ever been diagnosed with cancer?

0 No

1 Yes

99 Not sure

# Now, we would like to know how you feel about cervical cancer by asking the questions below:

# It is likely that I will get cervical cancer in the future

# 0 No

# 1 Yes

# 99 Not sure

# My chances of getting cervical cancer in the next few years are high

# 0 No

#

# 1 Yes

#

# 99 Not sure

# The thought of cervical cancer scares me

# 0 No

# 1 Yes

# 99 Not sure

# I am afraid to think about cervical cancer

# 0 No

# 1 Yes

# 99 Not sure

# Problems I would experience with cervical cancer would last a long time

# 0 No

# 1 Yes

#

# 99 Not sure

# Cervical cancer would harm a relationship with my partner

# 0 No

# 1 Yes

# 99 Not sure

# If I had cervical cancer my whole life would change

# 0 No

# 1 Yes

# 99 Not sure

# Now, we’d like to ask you some questions about health in general:

# I want to discover health problems early

# 0 No

# 1 Yes

# 99 Not sure

# Maintaining good health is important to me

# 0 No

# 1 Yes

# 99 Not sure

# I look for new information to improve my health

# 0 No

# 1 Yes

# 99 Not sure

# I have regular health check-ups even when I am not sick

# 0 No

# 1 Yes

# 99 Not sure

# It is important for my family to have good health

# 0 No

# 1 Yes

# 99 Not sure

# The next questions will help us understand how much you know about cervical cancer:

# I have heard of cervical cancer:

# 0 No

# 1 Yes

# 99 Not sure

# Human Papilloma Virus (HPV) causes cervical cancer:

# 0 No

# 1 Yes

# 99 Not sure

# It is possible to screen for cervical cancer:

0 No

1 Yes

99 Not sure

# A woman can have cervical cancer and not know it:

0 No

1 Yes

99 Not sure

# Screening for cervical cancer will find problems on the cervix before they turn into cancer:

0 No

1 Yes

99 Not sure

# These next questions will help us understand what you know about screening for cervical cancer:

# I want to be screened for cervical cancer:

0 No

1 Yes

99 Not sure

# I am afraid to be screened for cervical cancer for fear of a bad result:

# 0 No

# 1 Yes

# 99 Not sure

# I know how women are screened for cervical cancer:

0 No

1 Yes

99 Not sure

# I know where to go to be screened for cervical cancer:

# 0 No

# 1 Yes

# 99 Not sure

# Cervical cancer screening is safe:

0 No

1 Yes

99 Not sure

# Cervical cancer screening causes infertility:

0 No

1 Yes

99 Not sure

# Cervical cancer screening takes a long time:

# 0 No

# 1 Yes

# 99 Not sure

# Cervical cancer screening is painful:

0 No

1 Yes

99 Not sure

# All women should be screened for cervical cancer:

0 No

1 Yes

99 Not sure

# I would be screened for cervical cancer even if I had to pay for it:

# 0 No

# 1 Yes

# 99 Not sure

# I would tell my friends and family to be screened for cervical cancer:

0 No

1 Yes

99 Not sure

# If I had a problem on my cervix, I would return to the clinic to be treated:

0 No

1 Yes

99 Not sure

# I would agree to having a part of my cervix removed if that could fix the problem:

0 No

1 Yes

99 Not sure

# I would agree to having an operation if it was needed to fix the problem:

0 No

1 Yes

99 Not sure

# I want to know if I have cervical cancer:

0 No

1 Yes

99 Not sure

# My husband/family doesn’t mind if I am screened for cervical cancer:

0 No

1 Yes

99 Not sure

# My husband/family would allow me to have surgery if I needed it:

0 No

1 Yes

99 Not sure

# I would allow my daughter to have a vaccine for HPV if it helps to prevent cervical cancer:

0 No

1 Yes

99 Not sure

# I would allow my daughter to be screened for cervical cancer:

# 0 No

# 1 Yes

# 99 Not sure

# Finally we would like to ask some questions about you

# Date of birth __ __/___ __/___ ___ ___ ___

DD MM YY

1. Age ________ years
2. Educational level (Highest achieved)

0 None

1 Primary school

2 Secondary school

_3_University

# Relationship status:

0 single

1 married

2 widowed

3 divorced

4 partner, unmarried
